# The Effect of Physical-Activity-Based Programs on School Children’s Cognitive Competence-Related Variables: A Systematic Review of Randomized Controlled Trials

**DOI:** 10.3390/sports13080261

**Published:** 2025-08-08

**Authors:** Gorka Brioa Saez, Markel Rico-González, Natalia Monge Gómez

**Affiliations:** Department of Didactics of Musical, Plastic and Corporal Expression, University of the Basque Country, UPV-EHU, 48940 Leioa, Spain

**Keywords:** academic performance, cognition, physical education, primary education, systematic review

## Abstract

(1) Background: Aimed at improving school children’s motor, social, emotional, and cognitive competence (CC), the effects of physical activity (PA) have been widely investigated. However, investigations into the effects of PA during Physical Education (PE) on children’s CC have only been subject to growing interest among researchers in recent years. To bring it, the present article aims to systematically summarize the PE intervention programs whose effects on school children’s CC have been evaluated through a randomized controlled trial (RCT) design. (2) Methods: This systematic review follows the PRISMA guidelines and has been registered in PROSPERO: CRD420251083924. A systematic literature search was conducted across four electronic databases: PubMed, SCOPUS, Web of Science, and ProQuest Central. The articles were included based on the following inclusion criteria: (i) primary education children, (ii) children practicing PE, (iii) outcomes related to CC, and (iv) articles providing evaluations through RCTs. The quality assessment of all included articles was performed using the PEDro scale. (3) Results: Out of 219 initially identified studies, 25 met the inclusion criteria and were synthesized qualitatively. (4) Conclusions: The main outcomes highlighted that PE has a positive effect on primary school children’s cognitive ability, including attention, creativity, memory, academic performance, and inhibitory control. Thus, elementary school teachers are encouraged to implement these intervention programs to foster children’s CC. However, different types of interventions should be analyzed to highlight their effects on different variables of CC.

## 1. Introduction

Physical education (PE) is focused on the importance of physical activity (PA) for health and well-being [1], and there is growing interest in highlighting the role of this subject to foster integral child development (cognitive, emotional, and social competence) [2,3]. In general, PE is aimed at promoting and facilitating a meaningful understanding among students of their bodies and abilities. To that end, it allows them to acquire diverse skills that guide them to develop naturally in their environment, uphold a high quality of life, enjoy leisure, and maintain enriching relationships with others [4]. From this perspective, motor, social, emotional, and cognitive development must be addressed in an integrated way considering children’s continuous and progressive exploration of the environment, their relationships with objects and other children, the environment, and the teacher, which may help to build children’s topological, spatial, and problem-solving skills [5].

Among these competencies, cognitive competence (CC) has recently experienced exponential growth in the scientific literature, maybe because it describes how to manipulate internal information, which is crucial for autonomous learning and metacognition, that is, the ability to reflect on one’s learning process and adjust it as necessary [6]. In detail, cognition refers to the ability to process information through perception, acquired knowledge (experience), and subjective characteristics, which allows children to evaluate the information they perceive from the environment. When talking about CC, different concepts can be found, such as CC, academic achievement, and executive functions. These are connected, but they do not refer to the same concept. Specially, CC means a person’s mental abilities, such as thinking, solving problems, and remembering. Academic achievement refers to the level of success in educational pursuits. CC is an essential skill that can influence academic achievement, among other factors. Finally, executive function refers to the cognitive skills needed to manage daily tasks, plan, and adapt to new situations [7,8,9].

In this area, developing CC from the elementary educational stage is crucial, especially for PE as a subject, which could generate different neurobiological mechanisms during its practice. In fact, all the physical activities that a child performs (i.e., running, jumping, playing) encourage the use of brain energy and promote the development and final configuration of the brain. For example, children’s PA increased blood flow reaches the brain, which improves cerebral oxygenation and means that children receive a greater supply of essential nutrients for the main organ of cognition. In addition, an improvement in oxygenation can boost cognitive function by facilitating greater neural activity and better communication between brain regions [10]. Secondly, PA can increase neurotrophic factors among children [11]. These factors are similar to growth hormones, but they have a specific impact on the brain and are vitally important in promoting the survival and growth of neurons, as well as creating new synaptic connections [12]. Exercise-induced neuroplasticity can significantly improve cognitive function in the short and long term [13]. Third, PA targets the excitation of the nervous system as a result of exercise, which involves the formation and release of essential neurotransmitters, such as dopamine and serotonin. These brain chemicals play a critical role in regulating the mood, motivation, and attention, which can impact children’s development, with an increase in their release during and after exercise immediately improving children’s cognitive performance [13].

In this regard, Bernal [14] highlighted three groups of components that are consequences of practicing sport activities. First, during exercise, an increase in the concentration of neurotransmitters is observed, which facilitates the transmission of information from one neuron to another, promoting the development of memory. Second, the number of neurotrophic factors increases: these are specific proteins that promote the survival of neurons and, thus, contribute to the maintenance of brain health. Third, supramolecular adaptations are initiated and articulated, which increases the number of neurons, blood capillaries, and neuronal connections. This process also develops various parts and tissues of the brain, such as the hippocampus, a brain structure that plays a fundamental role in memory and the ability to navigate in space [14].

However, to the best of the authors’ knowledge, no systematic review has summarized different PE programs, evaluated through a randomized controlled trial (RCT) design, that have tried to influence primary school children’s CC. Therefore, the present article aims to systematically summarize the PE intervention programs whose effects on school children’s CC have been evaluated through an RCT design.

## 2. Materials and Methods

### 2.1. Experimental Approach to the Problem

This systematic review was conducted following the PRISMA (Preferred Reporting Items for Systematic Reviews and Meta-Analyses) framework [15] in conjunction with established methodological recommendations for systematic reviews within the field of sport sciences [16]. The systematic review has been registered in PROSPERO (PROSPERO 2025 CRD420251083924).

### 2.2. Information Sources

To find pertinent papers published up until 18 January 2024, a thorough search approach was applied across four electronic databases: PubMed, ProQuest Central, SCOPUS, and Web of Sciences.

### 2.3. Search Strategy

To clearly define the research question, the PICOS framework was employed. Whenever feasible, the search was restricted to peer-reviewed scientific articles and specific languages, as detailed in exclusion criterion number six. The screening process was not blinded with respect to journal titles or author identities. The search strategy incorporated the following terms (see Table 1):

(school OR “primary education” OR “elementary education”) AND (“physical education”) AND (“executive function*” OR cogniti* OR “academic performance” OR “academic achievement” OR “academic outcome*” OR “academic readiness” OR concentration OR memory OR attention) AND (“randomized controlled trial*”)

### 2.4. Eligibility Criteria

One author recorded key data from each article (including title, authorship, publication date, and source database) into a Microsoft Excel spreadsheet. Duplicate entries were subsequently removed. The remaining records were then screened based on the predefined inclusion and exclusion criteria (see Table 1). Additionally, other pertinent studies not initially captured through the database search were assessed following the same procedure and, if eligible, were incorporated under the label included from “external sources”.

### 2.5. Data Extraction

A Microsoft Excel spreadsheet was used for data extraction, in accordance with the Cochrane Consumers and Communication Review Group’s suggested template. The spreadsheet was used to assess each chosen study’s eligibility in light of the inclusion and exclusion criteria. The full-text articles that were not included in the analysis were listed together with the precise justifications. The spreadsheet was used to systematically archive all of the records.

### 2.6. Risk of Bias Assessment

Additionally, the second version of the Cochrane risk-of-bias tool for randomized trials was used (RoB 2). The authors assessed different key domains: random sequence generation (1 item), allocation concealment (2 item), blinding of participants and personnel (item 3), blinding of outcome assessment (item 4), incomplete outcome data (item 5), selective reporting (item 6), and other bias (item 7) [17]. Each domain was evaluated and classified using low risk of bias (+), high risk of bias (-), or some concerns or unclear risk of bias (?). Two authors conducted RoB 2.0 assessments (G.B.S. and M.R.-G.), with disagreements resolved through discussion or consultation with a third author (N.M.G.).

## 3. Results

### 3.1. Identification and Selection of Studies

**Figure 1:**
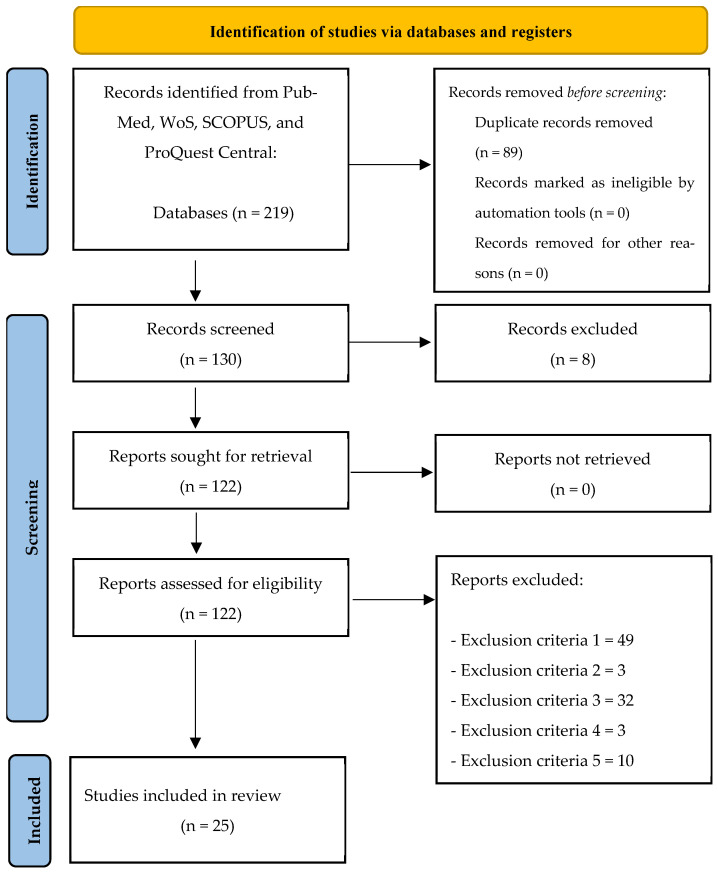
Flow diagram of the study.

A total of 89 of the 219 original papers (Proquest Central: 20; SCOPUS: 126; Web of Science: 37; PubMed: 36) were duplicates. As a result, 130 distinct articles were found. Eight papers were eliminated after titles and abstracts were reviewed because they did not satisfy the fifth inclusion criterion. The full text of the remaining 122 articles was then examined, and 49, 3, 32, and 3 articles were removed because they did not meet the exclusion criteria numbers 1, 2, 3, and 4. Additionally, 10 articles were not included because the programs were not evaluated through RCT design. The final qualitative synthesis, thus, contained 25 papers that satisfied all inclusion criteria (Figure 1).

### 3.2. Quality Assessment

Following the RoB-2 scale, the analysis provides that included studies assume risk of bias in some issue, mainly in allocation concealment and both blinding-related items: blinding of participants and personnel and blinding of outcome assessment. In fact, most of the articles declared that blinding was not performed during the interventions, assuming a high risk of bias due to these items. However, as the only RCTs were included in this systematic review, all studies met the first inclusion criteria. Finally, as different issues could provide risk of bias, all studies were classified as having some concerns in the items related to “other bias”. The results of the evaluated risk of bias are presented in Table 2.

### 3.3. Study Characteristics

The included studies were divided depending on the variables that were analyzed. They were divided into attention, inhibition, memory, cognitive flexibility, academic performance (mathematics, linguistics, and reading and writing), and creativity.

- Attention: Six studies evaluated the effects of a program on schoolchildren’s attention. Among them, four studies reported positive effects of intervention programs on children’s attention. Specifically, the Active-Start program revealed significant improvement in attention and concentration when cardiorespiratory fitness was at least, between 3.05 and 0.70 mL/kg/min, respectively [18]; the Movement Games program (based on MVPA) improves attention ability [19]; the external focus-based program showed more effects on attentional focus than internal focus-based exercises [32]; and a nuanced prediction of individual DMA indices spatial attention ability [33]. However, two studies did not find statistically significant effects of exercises based on aerobic efforts or cognitively demanding tasks when compared to usual PE classes [40] or acute PA on lapses of attention [37].
- Inhibitory control: Six studies evaluated the effects of a program on schoolchildren’s inhibition. Among them, one study demonstrated positive effects of the Movement Games intervention program compared with usual PE classes [19], while three studies demonstrated the effects of PA on children’s inhibition but did not find differences between groups. Specifically, Schmidt et al. [20] did not find more positive effects in different types of exercises (high cognitive engagement + high physical exertion, high cognitive engagement + low physical exertion, or low cognitive engagement + low physical exertion); Oppici et al. [29] did not find differences between high cognitive physical activity, low cognitive physical activity, or usual PE classes; and Kolovelonis & Goudas [31] did not find different effects of PA games highlighting contextual interference, mental control, or discovery. However, two studies did not demonstrate any effects on inhibition function [28,37].
- Memory: Six studies evaluated the effects of a program on schoolchildren’s memory capacity. Among them, three studies showed positive effects, while the other three did not demonstrate between-group differences. Specifically, high cognitive PA intervention improves memory better than low cognitive or habitual PE classes [29]; children performing PACER task intervention achieved better recall of words after a brief delay than children without treatment [26]; and children in physical activity games highlighting both contextual interference and mental control significantly enhanced working memory [31]. Meanwhile, other studies demonstrate how aerobic exercise or cognitively demanding exercises did not demonstrate evidence for effects on visuospatial short-term memory and verbal working memory [21]; external and internal focus instruction-based programs did not find the prediction of learning based on working memory capacity [32]; and integrating juggling with math lessons did not show better results than children performing sedentary math lessons on math memorization performance [35].
- Cognitive flexibility: Eight studies evaluated the effects of a program on schoolchildren’s cognitive flexibility. Among them, three studies found positive effects of evaluated programs. Specifically, the Active Recess program has better positive effects than intervention without physical exercise [23]; the Movi-Kids intervention showed better outcomes than usual PE classes on cognitive changes [34]; and martial-arts-based intervention showed better results than habitual PE classes on children’s cognitive self-regulation [38]. Another two studies found positive effects, although no differences between groups were found. In this case, PA games highlighting contextual interference, mental control, or discovery seem equally effective for improving cognitive flexibility when comparing with the no PE intervention group [31], In addition, Oppici et al. [29] support that high cognitive intervention could enhance cognitive flexibility, but they did not find statistical differences between groups (high cognitive or low cognitive PA). However, ball-skill-based intervention [22] and high-intensity interval training did not show better strategy than usual PE classes [30]; and the Active Smarter Kids program [28] did not show any effects on children’s cognitive flexibility.
- Academic performance: Twelve studies evaluated the effects of a program on schoolchildren’s academic performance. Among them, the Active-Start program showed positive effects on language and mathematics [18]; karate-based intervention improved children’s academic achievement [41]; an intervention program improved mathematics scores and language scores more than children performing habitual PE classes [30]; an enriched PE program improves better fluency and flexibility better than conventional PE classes [33]; karate-based intervention seems particularly effective for children with psychosocial difficulties and low academic performance [41]; PE classes taught by specialists are more effective on children’s numeracy and writing [36]; and a martial-arts-based program improves mental math performance [38]. However, other programs did not report significant effects after conducting their intervention programs. Among them, there are no effects of the ball-skill-based intervention program on learning lags in reading and mathematics [22]; there are no effects of aerobic, cognitive engaging, or habitual PE classes on academic achievement, although children with lower performance in reading at baseline performed better in reading at post-test after performing cognitive engaging intervention program [25]; after SPARK program intervention the authors highlighted that engaging children in more time of physical not necessarily influence academic performance [27]; the Active Smarter Kids intervention did not significantly improve verbal fluency [28]; and activity bursts in the classroom seem no more effective than normal curriculum activities for improving reading and mathematics [39]. However, it is of interest to analyze depending on the areas of the measured variables: general academic achievement, mathematics, speech, and literacy skills.
- Creativity: Three intervention programs were found. All of them supported significant differences in favor of ball skill-based [22], active recess-based [23], and enriched PE-based programs [33].
- Brain activation: One study evaluated the brain activation [21]. The authors found that greater brain activation was greater after both aerobic-based and cognitive-engaging interventions, although no statistical differences were found after them.

The characteristics of studies, as well as the results and conclusion of each article, were extracted and are clustered in Table 3. In addition, the characteristics of evaluated intervention programs are extracted in the Supplementary Material Table S1.

## 4. Discussion

The present article aims to systematically summarize the intervention programs whose effects on school children’s cognitive competence have been evaluated through RCT design. The main findings revealed that PE is clearly related to cognitive ability, including attention, creativity, memory, academic performance, and inhibitory control.

### 4.1. Effects of EF on Attention in Primary School Students

Attention refers to the mechanism directly involved in the activation and functioning of the processes of selection, distribution, and maintenance of psychological activity [42]. The search for RCTs showed a total of six studies that have evaluated their intervention programs on primary school children’s attention.

A unique study compared only two types of interventions. In this case, Brocken et al. [32] showed how an external focus-based program showed more effects on attentional focus than internal focus-based exercises [32]. However, three out of six studies compared a certain intervention program performed by the experimental group (EXP), while the control group (CON) continued with the usual PE classes [18,19,33]. According to the results of all studies, the Active-Start program [18], the enriched PE program [33], and the Movement Games Intervention program [19] had positive results in serving primary school children compared to conventional schools. Interestingly, the study of García-Hermoso et al. [18] tried to relate cardiorespiratory fitness to attention and concentration, highlighting that these two cognitive variables are improved when cardiorespiratory fitness is at least, between 3.05 and 0.70 mL/kg/min, respectively [18]. However, caution should be taken into account as a wide body of scientific literature has tried to link the effects of Vo_2max_ and CC, but some authors have tried to suggest that the relationship between physical fitness and CC is not necessarily dose–response because other factors (e.g., genetics, nutrition, environmental factors) may also play a role [7,43].

Other studies have compared the effects of cognitive engaging PA [37,40]. First, Meijer et al. [40] divided the students into three groups: aerobic exercise (EXP1), rigorous cognitive exercise (EXP2), and conventional PE classes. The results did not show a significant influence on the participants’ attention processes, which occurred in Van Der Fels et al. [37]. The aerobic and cognitively engaging physical intervention also did not positively influence lapses of attention.

Therefore, the Active-Start, enriched PE, and Movement Games interventions are recommended when compared to usual PE classes for improving schoolchildren’s attention. However, there is no clear result about the effects of cognitively engaging PA on schoolchildren’s attention, at least with the duration of the included interventions (the longer intervention was 14 weeks, four lessons per week).

### 4.2. Effects of PE on the Inhibitory Control of Primary School Students

Inhibitory control refers to the mental processes responsible for controlling them voluntarily and involuntarily, as well as the ability to prevent the interference of inappropriate information with ongoing responses and eliminate previously relevant information, which may provide a certain short-term stimulus, but that is not useful for the current task to be performed [44]. The search for RCTs showed a total of six studies that analyzed the effects of PE on the inhibitory control of primary school children.

Two out of five studies compared an intervention program assigned to the EXP with regular PE classes assigned to the CON [19,28]. In the study carried out by Chou et al. [19], the “Movement games” program was carried out to move in different situations, respond to the speed, direction, and strength of movements, and control body movements when jumping, throwing, catching, dribbling, kicking, or passing. The results showed an improvement in the EXP in inhibitory control, especially in the tendency to interfere. In the second study, Aadland et al. [28] investigated the consequences of the “Active Smarter Kids” intervention: active lessons for Norwegian, mathematics, and English (3 times a week, 30 min each), breaks from daily PA in the classroom (5 min), and PA tasks to complete at home (10 min a day). The results did not show the effects of EXP on inhibition functions. As in the case of attention, Van Der Fels et al. [37] stated that they found no influence on the inhibitory control of primary school students after an intervention based on aerobic exercises and another based on cognitively rigorous exercises.

On the other hand, three scientific studies compared three groups of children with interventions of different characteristics [20,29,31]. In the intervention carried out by Schmidt et al. [20], they divided the students into EXP1 (team games: high cognitive engagement + high physical exertion), EXP2 (aerobic exercises: low cognitive engagement + high physical exertion), and CON (low physical + cognitive demand program). The conclusion reached in this study is that there are no differences between the three groups and that the level of inhibitory control is the same as with CON. However, the study conducted by Kolovelonis and Goudas [31] showed differences in interventions based on PA games, mental-control-based exercises, and discovery-based exercises, compared to children who did not engage in physical activity. Finally, Oppici et al. [29] compared a high cognitive load intervention (EXP1) and a low cognitive load intervention (EXP2) with a group that continued with ordinary PE classes (CON). This study showed that although EXP1 could improve inhibitory control, more research is needed to study the generalizability of the findings.

In brief, the “Movement Games” program, exercises based on mental control, and exercises based on discovery have shown positive results in inhibitory control, while low physical and cognitive demand programs, sports programs, and the intervention in “Active Smarter Kids” had no positive effects. Exercises with high cognitive demand continue to be the subject of discussion.

### 4.3. Effects of PE on the Memory of Primary School Students

Memory refers to the psychological process that serves to store encoded information. This information can be recovered, sometimes voluntarily and consciously, and other times involuntarily [45]. The search for RCTs showed a total of six studies that analyzed the effects of PE on primary school children’s memory.

Some RCTs found no improvement in the effects of PE on children’s memory. For example, the “juggling intervention program”, which introduced juggling into mathematical practice, did not show improvements in multiplication memorization performance [35]. Likewise, in the research carried out by Meijer et al. [40], aerobic exercises, highly cognitive exercises, or conventional PE classes were proposed, but improvements in memorization were not found either.

However, there are two studies that do not correspond to the previous results, showing improvements in memory capacity and working memory. In the first of them, Oppici et al. [29] carried out two interventions related to learning dance choreography: EXP1, which carried out an intervention with high cognitive content, and EXP2, which carried out an intervention with low cognitive content. In the second study, Kolovelonis and Goudas [31] compared three types of PA games, cognitively challenging the students’ memory capacity, while CON did not participate in PE. EXP1 played games that highlighted context interference, EXP2 played games that highlighted mental control, and, finally, EXP3 played games that highlighted discovery. In both they found improvements in all EXP. Furthermore, findings from various interventions suggest that rigorous exercise provides benefits to long-term memory [26] and that older children have greater memory capacity than younger children [32].

In summary, the scientific literature shows that exercise can improve memory, but results vary depending on the type of intervention and its cognitive content. In general, interventions with high cognitive content appear to be more effective in improving memory capacity and working memory. However, future studies must corroborate these facts, which, in the absence of further analysis, have adverse effects.

### 4.4. The Effects of PE on the Cognitive Flexibility of Primary School Students

Cognitive flexibility refers to the ability to adapt functions to environmental conditions when faced with a task or as an integral part of executive functions [46]. The search for RCTs showed eight studies that analyzed the effects of PE on the inhibitory control of primary school children.

The “Active recess program” [23], the “Movi-Kids” [34], and the “LEAD” [38] programs showed notable improvements in cognitive flexibility. These programs compared the intervention to a CON group. In the case of “Movi-Kids” and “LEAD”, the students continued with the usual PE classes, while in the case of the “Active recess program”, the students carried out usual activities in class without physical exercise. Likewise, Kolovelonis and Goudas [31] compared the effects of three different types of PA games that challenge cognitive flexibility with a CON who did not participate in PE. The results of this study also revealed the positive effects of cognitively challenging physical activities, which highlighted context interference and mental control.

However, four studies found no relationship between the interventions carried out and cognitive flexibility [22,28,29,30]. Specifically, Westendorp et al. [22] carried out an intervention to exercise with the ball, while the CON group followed the usual PE classes. Likewise, Aadland et al. [28] performed the Active-Start Kids (ASK) intervention, while CON continued with the usual PE classes. Oppici et al. [29] divided them into two intervention groups: EXP1 (high cognitive load), EXP2 (low cognitive load), and CON, as in the other two studies, followed by the usual PE classes. Finally, Takehara et al. evaluated the effects of high-intensity interval training compared with usual PE classes [30]. As mentioned above, none of these studies had positive results in cognitive flexibility.

Consequently, studies on the impact of PE on cognitive flexibility showed mixed results. While some programs noticeably improved this ability, others found no connection, highlighting the need for more research to better understand these findings.

### 4.5. Effects of PE on the Academic Performance of Primary School Students

Academic performance corresponds to the level of knowledge demonstrated in an area or subject in comparison to the norm of age and academic level [47]. Academic performance can be divided into several variables (e.g., academic performance in mathematics, speaking, reading, and writing). The search for RCTs showed a total of 12 studies that analyzed the consequences of PE on the academic performance of primary school children. However, within this competency there are numerous variables measured: general academic achievement, mathematics, speech, and literacy skills.

Without specifying the cognitive performance variable, Pinto-Escalona et al. [24,41] conducted two studies. In these studies, the sample of children was distributed in an EXP group that carried out an intervention based on karate-related exercises, while in CON they continued with the usual PE classes. In both studies they found benefits in academic performance in favor of EXP compared to CON. However, the rest of the studies determined the variable they wanted to measure within academic performance.

#### 4.5.1. Academic Performance in Mathematics

Different studies investigated the academic performance variable in mathematics. All these studies proposed the comparison of a program designed for EXP, and the CON group followed the usual PE classes [18,22,27,30,36,38,39]. Active-Start [18], the ball skills program [22], SPARK [27], “Activity Bursts in the Classroom” or ASK [28], exercise intervention [30], GH [36], and LEAD [38], taught by specialists, showed notable improvements in mathematical competence compared to regular classes from EF. Although most studies seem to agree that programs to improve mathematical competence are more appropriate than conventional classes, there are two that oppose this. Specifically, children who participated in the “Activity Bursts in the Classroom” program [39] did not show better results than those who followed the usual PE classes. Likewise, De Bruijn et al. [25] compared three types of children’s groups in three interventions: aerobic exercises (EXP1), the cognitively stimulating program (EXP2), and the program based on low cognitive and physical demand (CON). Therefore, although in the primary school boy and girl mathematical competence seems to be a clear trend towards the positive effects of PE (the Active-Start programs, the ball skills program, SPARK, ASK, exercise intervention, PE, and LEAD provided by specialists), some details must be analyzed, such as the duration and what type of activity has been proposed, as both studies have not found improvements.

#### 4.5.2. Academic Performance in Speaking

The four investigations analyzed the variable academic performance of speaking ability through the execution of various programs [18,28,30,48]. The Active-Start proposal contains physically active educational subjects, brain breaks, and PA obligations [18]. On the other hand, the exercise intervention is an activity based on high-intensity interval training (HIIT) accompanied by music, which is divided into four parts with the aim of improving aerobic fitness and motor skills [30]. Both studies showed beneficial results of PE on language ability. On the contrary, the ASK [28] and the Active Values [48] programs did not show significant differences in verbal fluency, as they compared EXP and CON. Therefore, although some programs appear to show positive results of PE on primary school children’s speech (the Active-Start and the HIIT programs), in general, there is debate over the results shown, suggesting the need for new randomized controlled analyses.

#### 4.5.3. Academic Performance in Reading and Writing

Finally, five studies investigated the variable academic performance in the aspect of literacy [22,25,27,36,39]. The ball training programs [22], SPARK [27], the GH program taught by specialists [36], and “Activity Bursts in the Classroom” [39] compared their program with a CON, and the participating children continued with the previously established programming. For their part, De Bruijn et al. [25] compared the programs carried out by the author with three groups of different characteristics: the aerobic intervention (EXP1), the cognitively rigorous program (EXP2), and the intervention based on low physical and cognitive demand (CON). All studies concluded that the programs carried out have no impact on reading. However, Telford et al. [36] related PE classes taken, taught by specialists, with greater improvements in writing. Therefore, the findings obtained indicate that there is no correlation between PE and academic performance in literacy, although the participation of experts in the subject could show a thread to follow in future research.

### 4.6. The Effects of PE on the Creativity of Primary School Students

Creativity refers to the ability to create new ideas or concepts, innovate, create, or create new links between known ideas and concepts, which normally lead to new consequences, solve problems, and create original and valuable solutions [49,50]. The search for RCTs showed a total of three studies that examined the effects of PE on elementary school children’s creativity.

Latorre-Roman et al. [23] conducted a study to evaluate the effects of an active leisure program on creativity. In this study, 114 children were divided into two groups: the EXP, which carried out the active play program, and the CON, which followed the usual PE classes. The results showed a greater increase in all creative measures compared to the CON. Likewise, a study was conducted by De Fano et al. [33] to investigate the originality, where 242 children participated. The EXP participated in the enriched PE, while the CON taught regular PE classes. The results showed that EXP can provide short-term benefits for the originality of children’s motor behavior, but additional studies are needed to understand the long-term effects on this variable. These positive results remain in children with special educational needs. In this sense, Westendorp et al. [22] conducted a study to determine the influence of an intervention based on ball skills exercises in problem solving of children with learning disabilities. The EXP received an intervention program to train with the ball, while the CON program received the usual PE classes. A positive relationship was found between improving ball skills and improving EXP problem solving.

Therefore, the scientific studies unanimously show that PE has positive effects on primary school students’ creativity, both in children without special educational needs and in children with a learning disorder. However, additional studies are suggested to corroborate these findings.

## 5. Limitations

To analyze articles with a lower risk of bias, there were only RCTs included in the present systematic review. However, the RoB-2 scale was used to analyze them, and some issues should be highlighted. Mainly, the extracted results should be taken with caution because items such as allocation concealment and blinding-related items may influence the overall findings. In fact, although blinding participants, assessors, or researchers that will perform the assessment is a common limitation in educational studies that have included young children, the absence of blinding increases the likelihood of performance and detection bias, which may overestimate the reported effects of the interventions.

Additionally, it is important to highlight that due to the wide variety of intervention programs, as well as the different evaluated variables, it is difficult to highlight and generalize what is the most suitable type or intensity of exercise to improve each of the different cognitive-related variables.

## 6. Conclusions

Overall, PE has positive effects on the cognitive abilities of primary school students, especially with specific programs with high cognitive demand. Although the benefits are based on different areas, PE’s impact on some skills shows mixed results, indicating the need for more research to optimize interventions. In general, PE contributes significantly to the cognitive development of students. However, this competition has been divided into several sections, based on the findings:

For attention, Active-Start, enriched PE, and the Movement Games intervention show improvements in care for school-aged children. However, high cognitive demand programs and aerobic exercises do not seem to positively affect child attention.

Regarding inhibitory control, Movement Game programs, mental control exercises, and discovery-based activities have shown positive results in inhibitory control. On the contrary, programs with low physical and cognitive demand, sports programs, and the “Active Smarter Kids” intervention did not have positive effects. The scientific literature continues not to support those exercises with high cognitive demand for improving CC.

For improving memory, evidence indicates that exercise can improve memory, but that results depend on the type of intervention and its cognitive content. In general, interventions with high cognitive content appear to be more effective in improving memory capacity and working memory. However, more research is necessary to confirm these findings, as the current conclusions are contradictory due to a lack of sufficient studies.

After checking articles that have analyzed cognitive flexibility, a wide variety of results can be found. Some programs showed significant improvements in this skill, while others did not find relationships, underscoring that more research is needed to better understand these results.

However, when checking outcomes related to academic performance, we can extract general conclusions and other specifics by analyzing each sub-variable within it. As a general conclusion, it should be noted that the impact of PE on the academic performance of primary school boys and girls has very varied results. Some interventions have seen notable improvements, but others have had no positive effects. The variability of the results suggests that factors such as the type of program and its specific content may influence the observed benefits. After this general conclusion, a conclusion can be highlighted for mathematical, linguistic, and reading and writing competencies. Mathematical competence: In the mathematical competence of primary school children there is a clear trend towards the positive effects of PE, in which the Active-Start, ball skills, SPARK, ASK, exercise intervention, PE taught by specialists, and LEAD programs stand out. It is essential to analyze some details, such as the duration and the specific type of activity proposed. The reason is that both studies did not observe substantial improvements in this aspect, suggesting that the effectiveness of PE may depend on factors such as the duration of the program and the type of activity included. Linguistic competence: Although some programs such as the Active-Start and the HIIT generate favorable effects of PE on the language of school-aged children, in general, a discussion is observed in the results presented. This underscores the need for new randomized controlled studies to more consistently clarify and validate these findings. Reading and writing: The results obtained suggest that there is no direct relationship between PE and academic activity on schoolchildren’s literacy. However, the involvement of experts could be an important starting point for future research.

Finally, when checking articles that have evaluated the effects of PE on children’s creativity, the scientific studies reviewed show a consensus regarding the positive effects of PE on primary school students’ creativity, including children without special educational needs and those with learning disorders. However, there is a need for additional research to protect and validate these findings more broadly and decisively.

## Figures and Tables

**Table 1:** Eligibility criteria for the inclusion and exclusion of studies.

PICOS			Inclusion	Exclusion	Search Coherence
1	P	Population	Children from elementary school.	Children who do not attend elementary school.	(school OR “primary education” OR “elementary education”)
2	I	Intervention or Exposure	Children participating in physical education during school hours.	Children not participating in physical education during school hours. Intervention related to virtual reality.	“physical education”
3	C	Comparison	-	-	-
4	O	Outcome(s)	Outcomes related to cognition.	Results extracted from non-objective measures: teacher’s opinion, interviews, observations, perceptions, or experiences during a certain program. Program proposals without considering children in their studies. Study protocols.	“executive function*” OR cogniti* OR “academic performance” OR “academic achievement” OR “academic outcome*” OR “academic readiness” OR concentration OR memory OR attention
5	S	Study design	Randomized controlled trials.	Non-randomized controlled trials.	“randomized controlled trial*”
6	-	Other criteria	Peer-reviewed, original, full-text studies, written in English or Spanish.	Written in another language or without peers, reviewing the complete original text studies.	-

**Table 2:** Risk of bias using RoB-2.

Study	Random Sequence Generation	Allocation Concealment	Blinding of Participants and Personnel	Blinding of Outcome Assessment	Incomplete Outcome Data	Selective Reporting	Other Bias
Garcia-Hermoso et al. [18]	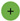	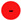	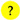	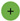	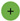	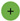	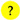
Chou et al. [19]	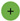	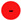	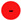	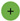	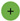	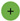	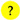
Schmidt et al. [20]	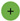	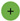	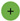	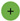	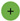	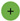	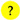
De Bruijn et al. [21]	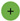	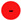	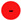	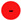	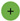	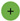	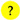
Westendorp et al. [22]	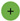	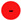	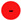	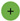	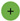	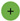	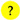
Latorre- Román et al. [23]	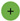	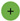	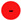	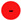	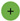	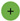	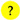
Pinto-Escalona et al. [24]	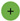	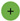	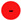	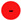	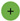	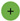	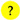
De Bruijn et al. [25]	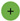	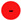	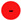	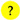	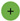	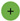	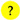
Etnier et al. [26]	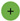	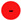	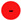	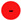	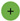	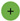	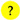
Sallis et al. [27]	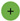	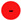	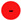	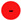	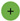	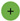	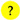
Aadland et al. [28]	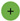	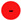	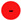	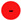	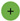	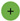	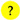
Oppici et al. [29]	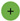	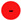	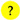	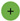	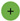	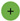	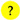
Takehara et al. [30]	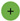	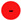	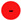	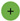	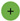	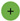	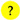
Kolovelonis & Goudas [31]	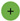	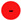	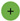	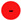	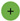	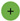	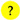
Brocken et al. [32]	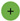	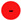	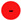	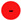	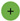	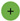	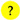
De Fano et al. [33]	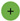	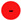	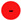	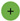	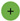	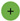	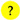
Sánchez-López et al. [34]	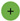	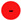	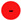	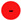	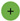	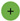	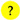
Van Den Berg et al. [35]	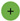	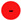	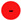	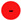	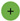	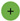	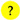
Telford et al. [36]	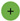	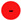	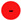	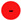	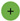	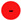	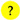
Van Der Fels et al. [37]	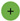	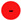	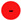	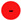	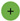	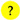	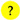
Lakes & Hoyt. [38]	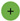	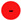	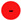	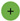	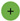	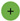	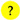
Katz et al. [39]	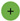	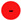	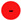	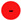	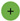	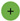	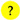
Meijer et al. [40]	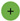	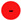	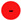	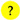	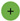	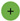	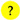
Pinto-Escalona et al. [41]	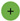	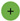	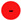	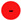	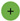	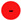	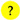

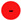
 High risk, 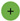 Low risk, 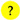 Some concerns.

**Table 3:** Characteristics of included studies.

Reference	Aim	Sample	Intervention			Results			Conclusions
			Name/Groups	Intervention	Duration	Test Instrument	Variables	Results	
Attention									
García-Hermoso et al. [18]	Analyze the implementation of a before school PE classes in concentration and attention ability.	Nº children: 171. Schools: 3. Country: Chile. Age: 8−10.	EXP (n = 100) Active-Start	EXP did Active-Start program while CON did usual PE classes.	8 weeks 5 times per week.	d2 Test of Attention.	Selective attention. Concentration capacity.	Although significant changes were not found in attention and concentration, the study revealed significant improvements in these variables when cardiorespiratory fitness was, at least, 3.05 and 0.70 mL/kg/min, respectively.	Purpose a PA program that enhance Vo2_max_ before school classes may foster attention and concentration.
			CON (n = 70) Usual PE.						
Chou et al. [19]	Evaluate the effects of a program on cognitive-related variables among overweight children.	Nº children: 84. Schools: 4. Country: Taiwan. Age: 10−12.	EXP (n = 44) Movement Games intervention.	EXP did Movement Games intervention program while CON did usual PE classes.	8 weeks 3 times per week.	Stroop test.	Attention function.	Response accuracy rate: significant time effect in EXP (F(1, 43) = 71.45, p 01, partial η^2^ = 0.62 with moderate ES, whereas no change was noted in the CON, F(1, 39) = 3.24, *p* > 0.05). Response accuracy rate higher in EXP. Correct on time: significant time effect in EXP (F(1, 43) = 88.94, *p* < 0.001, partial η^2^ = 0.67 with moderate ES, but not in the control group, F(1, 39) = 1.67, *p* > 0.05). In addition, a significant group effect was noted in the post-test, t(82) = −6.04, *p* < 0.001, d = 0.84 with moderate to high ES.	Movement Games Intervention, involving children in MVPA, is suitable for improving attention abilities in overweight children.
			CON (n = 40) Habitual PE classes.						
Brocken et al. [32]	Explore the effects of age on attentional focus on motor learning.	Nº children: 60. Schools: doesn’t say. Country: Age: 8−12.	EXP1 (n = 30) External focus instructions	EXP1 did external focus instructions. while EXP2 did internal focus instruction.	2 sessions.	AWMA.	Attentional focus.	Effects of attentional focus instruction on motor learning: the EXP1 achieved better results than EXP2. In detail: the pretest score was a significant covariate, F(1, 55) D 30.96, *p* < 0.001, h2 D 0.36. A main effect of instruction was found, F(1, 55) D 7.29, p D 0.009, h2 D 0.12, indicating that the EXP1 showed larger improvements in putting performance than the EXP2. No significant main effect of age, F(1, 55) D 1.06, p D 0.31, partial h2 D 0.02, and no significant interaction between age and instruction, F(1, 55) D 0.33, p D.57, partial h2 D 0.006.	External focus-based program is more effective than internal focus-based program.
			EXP2 (n = 30) Internal focus instructions.						
De Fano et al. [33]	To assess the attentional predictors of divergent movement ability (DMA) in childhood and the role of sport and enriched PE experience.	Nº children: 242. Schools: 2. Country: Italy. Age: 10−11.	EXP (n = 129) Past enriched PE.	EXP did past enriched PE while CON did usual PE classes.	6 months 1 time per week 1 h.	Line-bisection task.	Attentional skills.	Through regression analysis, the outcomes showed a nuanced prediction of individual DMA indices spatial attention ability.	The results identify novel cognitive determinants of children’s DMA.
			CON (n = 112) Past conventional PE.						
Meijer et al. [40]	To examine the effects of two 14-week school-based exercise interventions on neurocognitive functioning.	Nº children: 93. Schools: 22. Country: The Netherlands. Age: 7−11.	EXP1 (n = 30) Aerobic exercise.	EXP1 did aerobic exercise and EXP2 did cognitively demanding exercise while CON did habitual PE classes.	14 weeks 4 lesson per week.	WISC-III.	Alerting attention. Information processing. Spatial attention.	The results did not show statistical significative effects after both EXP1 or EXP2.	14-week PA program based on aerobic exercise or cognitive-demanding exercise seems not effective enough to influence brain structure and brain function.
			EXP2 (n = 32) Cognitively demanding exercise.						
			CON (n = 31) Habitual PE classes.						
Van Der Fels et al. [37]	To assess the effectiveness of a program on children’s lapses of attention.	Nº children: 89. Schools: 24. Country: The Netherlands. Age: 8−10.	EXP (n = 43) Aerobic PA and Cognitively engaging PA.	EXP did aerobic PA and cognitively engaging PA intervention while CON did regular classroom lessons.	2 days.	Performed pretest. Performed post-test.	Lapses of attention.	After PA intervention, the results did not show improvements in lapses of attention.	Acute PA did not have positive effects on lapses of attention.
			CON (n = 47) Regular classroom lessons.						
**Inhibition**									
Chou et al. [19]	To examine the effects of a program on overweight children’s executive function.	Nº children: 84. Schools: 4. Country: Taiwan. Age: 10−12.	EXP (n = 44) Movement Games intervention.	EXP did Movement Games intervention program while CON did usual PE classes.	8 weeks 3 times per week.	Determination test.	Inhibitory control.	Mainly in the interference tendency condition, children that performed program of movement games showed improvements in inhibitory control, while no performance improvements was noted in the original PE lessons.	The program performance by children in EXP is suitable for improving inhibitory control.
			CON (n = 40) Habitual PE classes.						
Schmidt et al. [20]	To compare the effects of two different programs on executive functions.	Nº children: 181. Schools: 12. Country: Switzerland. Age: 10−12.	EXP1 (n = 69) Team games (high cognitive engagement, high physical exertion)	EXP1 did team games program, EXP2 did aerobic exercise program, and CON did a program based on low cognitive and low physical exertion.	6 weeks 2 times per week.	Flanker task.	Inhibitory control.	Although the results showed that the data significantly better for inhibition, χ^2^ (1, N = 181) = 4200.36−4195.86 = 4.50, *p* < 0.05, it did not differ significantly between groups (F(2, 178) = 0.06, *p* = 0.947).	PA can affect executive function, although the change in inhibition did not differ between three different interventions.
			EXP2 (n = 57) Aerobic exercise (low cognitive engagement, high physical exertion).						
			CON (n = 55) Control condition (low cognitive engagement, low physical exertion).						
Oppici et al. [29]	To evaluate different dance choreography-based different interventions affects executive functions.	Nº children: 74. Schools: 1. Country: Australia. Age: 8−10.	EXP1 (n = 30) High-cognitive intervention.	EXP1 did high-cognitive intervention, EXP2 did low-cognitive, and CON did usual PE classes.	7 week. 2 lessons per week.	Flanker test.	Inhibitory control.	The results showed a statistically significant time effect (F(1,73) = 10.44, *p* < 0.01, ηp^2^ = 0.13) and no significant effect of group (*p* = 0.69) nor group time (*p* = 0.33). Pairwise comparisons showed a significant pre-to-post improvement in the CON group only (T [18] = 3.3, *p* < 0.01, D = 0.33 ± 0.21, d = 0.41).	Although initially the results showed that high-cognitive intervention support that it could improve inhibition control, not statistically significant differences between groups highlighted the importance of future research.
			EXP2 (n = 29) Low-cognitive group.						
			CON (n = 19) Habitual PE classes.						
Van Der Fels et al. [37]	To analyze the effects of acute exercise and cognitive engaging exercise on response inhibition and lapses of attention.	Nº children: 89. Schools: 24. Country: The Netherlands. Age: 8−10.	EXP (n = 43) Aerobic PA and cognitively engaging PA.	EXP did aerobic PA and cognitively engaging PA intervention while CON did regular classroom lessons.	2 days.	Performed pretest. Performed post-test.	Response inhibition. Lapses of attention.	Regarding acute PA vs. seated classroom lesson, the study did not show significant improvements in the covariates model for response inhibition, Δχ^2^(1) = 0.08, *p* = 0.77, lapses of attention, Δχ^2^(1) = 0.97, *p* = 0.33, mean reaction time, Δχ^2^(1) = 0.46, *p* = 0.50, and μ, Δχ^2^(1) = −0.01, *p* = 0.91. Regarding aerobic vs. cognitively engaging PA, the study did not show significant improvements in the covariates model for response inhibition, Δχ^2^(2) = 0.07, *p* = 0.97, lapses of attention, Δχ^2^(2) = 0.91, *p* = 0.63, mean reaction time, Δχ^2^(2) = 3.69, *p* = 0.16, and μ, Δχ^2^(2) = 3.12, *p* = 0.21.	The acute exercise and these cognitive functions following the PA program did not show positive effects on response inhibition and lapses of attention.
			CON (n = 47). Regular classroom lessons.						
Aadlamd et al. [28]	To evaluate the effects of curriculum prescribed program on executive functions.	Nº children: 1202 Schools: 57 Country: Norway Age: 10.	EXP (n = 596) Active Smarter Kids.	EXP did Active Smarter Kids intervention while CON did usual PA classes.	7 months 135 min per week of PA.	Stroop test.	Inhibition.	The outcomes did not demonstrate any effects on inhibition function (mean group difference 0.2–1.2%, 0.01–0.06 SD units, *p* = 0.191−0.893) of children involved in EXP.	The intervention did not show that is a suitable option to increase executive functions.
			CON (n = 533) Habitual PE classes.						
Kolovelonis & Goudas [31]	To evaluate the effects of different intervention programs on executive functions.	Nº children: 140. Schools: 4. Country: Greece. Age: 11.	EXP1 (n = 29) PA games highlighting contextual interference.	EXP1 did PA games highlighting contextual interference, EXP2 did PA games highlighting mental control, EXP3 PA games highlighting discovery did, and CON did not participate in PE.	3 45-min sessions per week	Design fluency test.	Inhibition.	Children from all EXP groups showed significative positive effects on inhibition compared to CON.	All intervention programs are effective to improve inhibition functions.
			EXP2 (n = 36) PA games highlighting mental control.						
			EXP3 (n = 36) Physical activity games highlighting discovery.						
			CON (n = 39) No PE .						
**Memory**									
Maijer et al. [40]	To analyze the effects of an exercise interventions on neurocognitive functioning.	Nº children: 93. Schools: 22. Country: The Netherlands. Age: 7−11.	EXP1 (n = 30) Aerobic exercise.	EXP1 did aerobic exercise and EXP2 did cognitively demanding exercise while CON did habitual PE classes.	14 weeks 4 lesson per week.	WISC-III.	Visuospatial short-term memory. Verbal working memory.	There is no evidence for effects on visuospatial short-term memory and memory verbal working memory.	This study indicated that aerobic exercise or cognitive demanding exercise not necessarily improve memory.
			EXP2 (n = 32) Cognitively demanding exercise.						
			CON (n = 31) Habitual PE classes.						
Oppici et al. [29]	To evaluate different dance choreography-based different interventions affects executive functions.	Nº children: 74. Schools: 1. Country: Australia. Age: 8−10.	EXP1 (n = 30) High-cognitive intervention.	EXP1 did high-cognitive intervention, EXP2 did low-cognitive, and CON did usual PE classes.	7 week 2 lessons per week.	Working memory test DCSS.	Memory capacity.	EXP1 children’s working memory significantly improves (*p* < 0.01; d = 0.51), while the low-cognitive (*p* = 0.04; d = 16 0.48) and control groups did not (*p* = 0.32; d = 0.17). However, difference between groups was not statistically significant.	Although outcomes of high-cognitive intervention support that it could improve working memory, not statistically significant differences between groups highlighted the importance of future research.
			EXP2 (n = 29) Low-cognitive group.						
			CON (n = 19) Habitual PE classes.						
Etnier et al. [26]	To compare the effects of an acute bout of exercise on learning, short-term memory, and long-term memory.	Nº children: 43. Schools: 1. Country: United States. Age: 11−12.	EXP (n = 24) PACER task intervention.	EXP did PACER task intervention program while CON did no-treatment control condition exercise.	2 days.	RAVLT.	Short- and long-term memory.	Checking the word list, children in EXP achieved significantly better recall of the words after a brief delay.	This study supports acute bout of exercise to provide benefits for verbal learning and long-term memory.
			CON (n = 19) No-treatment control condition.						
Kolovelonis & Goudas [31]	To evaluate the effects of different intervention programs on executive functions.	Nº children: 140. Schools: 4. Country: Greece. Age: 11.	EXP1 (n = 29) PA games highlighting contextual interference.	EXP1 did PA games highlighting contextual interference, EXP2 did PA games highlighting mental control, EXP3 PA games highlighting discovery did, and CON did not participate in PE.	3 45-min sessions per week.	Design fluency test.	Memory capacity.	Comparing to CON, children in PA games highlighting both contextual interference (EXP1) and mental control (EXP2) significantly enhanced working memory. These games required students to engage in non-repeating, changing sequences of actions and to hold and manipulate information while reacting to signals. Such conditions likely prompted increased mental effort and rehearsal, contributing to improvements in working memory.	Non-repeating, changing sequences of actions and to hold and manipulate information while reacting to signal are suitable strategies for implementing during PA exercises for improving memory.
			EXP2 (n = 36) PA games highlighting mental control.						
			EXP3 (n = 36) PA games highlighting discovery.						
			CON (n = 39) No PE were involved.						
Brocken et al. [32]	Explore the effects of age on attentional focus on motor learning.	Nº children: 60. Schools: - Country: - Age: 8−12.	EXP (n = 30) External focus instructions	EXP did external focus instructions while EXP2 did internal focus instruction.	2 sessions.	AWMA.	Working memory capacity.	Younger children had lower verbal working memory ability than older children younger children, whereas no significant differences between groups existed. Age: a large main effect (F(1, 56) D 10.6, *p* < 0.01, partial h2 D 0.16), but not of instruction, F(1, 56) D 0.84, p D 0.36, partial h2 D 0.02). No interaction between age and instruction was found, F(1, 56) D 0.05, p D 0.83, partial h2 D 0.001.	In external and internal focus, instruction-based programs do not found the prediction of learning based on working memory capacity.
			EXP2 (n = 30) Internal focus instructions.						
Van Den Berg et al. [35]	To assess the effectiveness of integrating juggling with math classes on multiplication memorization performance.	Nº children: 323. Schools: 9. Country: The Netherlands. Age: 10.4.	EXP (n = 170) Juggling intervention program.	EXP did juggling intervention program while CON did control program.	5 weeks 20 sessions 5 to 8 min	CITO test battery A multiplication tables test.	Math memorization performance.	Children in EXP enjoyed more than children in sedentary math group. However, no significant effect were found on multiplication memorization performance.	Although no significant improvements were found, the increased enjoyment in the math-juggling group can sed light on for structurally incorporating physical activities in the classroom setting.
			CON (n = 153) Control program.						
**Cognitive flexibility**									
Westendorp et al. [22]	To evaluate the effects of ball-skills-based program on cognitive parameters of children with learning disorders.	Nº children: 91 Schools: 1 Country: The Netherlands Age: 7−11.	EXP (n = 45) ball skill intervention.	EXP did ball skill intervention program while CON did usual PE classes.	16 weeks 2 times per week 40 min each session.	TMT	Cognitive flexibility.	The results did not show any effects on cognitive parameters. No significant correlations (*p* > 0.05) between ball skills and cognitive flexibility during the intervention or 6 months after in both groups.	The program performed by children in EXP seem not effective to show improvements on cognitive parameters, at least, in children with learning disorders.
			CON (n = 46) Habitual PE classes.						
Latorre-Román et al. [23]	Evaluate the effects of active recess program on children’s cognitive flexibility.	Nº children: 114. Schools: 3. Country: Spain. Age: 8−12.	EXP (n = 58) Active Recess Program.	EXP did Active Recess program while CON did habitual class activities with no physical exercise	10 weeks 3 times per week 30 min each activity.	TMT	Cognitive flexibility.	The intervention program shows significant improvements on children’s cognitive flexibility. In fact, children in EXP increase their cognitive flexibility greater (*p* < 0.05) than the children in CON (*p* < 0.05).	The Active Recess program is suitable to foster cognitive flexibility.
			CON (n = 56) Habitual activities with no physical exercise.						
Aadlamd et al. [28]	To evaluate the effects of curriculum prescribed program on executive functions.	Nº children: 1,202 Schools: 57 Country: Norway Age: 10	EXP (n = 596) Active Smarter Kids.	EXP did Active Smarter Kids intervention while CON did usual physical educational classes.	7 months 135 min per week of PA.	TMT	Cognitive flexibility.	Following intention-to-treat analysis, children in EXP did not show any effects on their executive functions (mean group difference 0.2–1.2%, 0.01–0.06 SD units, *p* = 0.191–0.893). However, by conducting supplementary per protocol analyses, the authors obtained small significant effects of the intervention on the composite score of executive functions and cognitive flexibility.	Cognitively engaging and coordinative demanding activities/games could be viable options to improve executive functions and, it is possible that it can improve academic performance.
			CON (n = 533) Habitual PE classes.						
Oppici et al. [29]	To evaluate different dance choreography-based different interventions affects executive functions.	Nº children: 74. Schools: 1. Country: Australia. Age: 8−10.	EXP1 (n = 30) High-cognitive intervention.	EXP1 did high-cognitive intervention, EXP2 did low-cognitive, and CON did usual PE classes.	7 week 2 lessons per week.	DCSS Flanker test.	Cognitive flexibility.	ANOVA showed a statistically significant time effect (F(1, 73) = 9.84, *p* < 0.01, ηp^2^ = 0.13), and no significant effect of group (*p* = 0.30) nor group time (*p* = 0.53) in the DCSS score. Pairwise comparisons did not show any statistically significant improvement in the three groups. The exploratory ANOVA showed a significant effect of time (F(1, 73) = 9.70, *p* < 0.01, ηp^2^ = 0.13). For the within-group pairwise comparisons, ANOVA showed no significant effects in all three groups. T-test showed that the males significantly improved their score (T[1,11] = 2.20, *p* = 0.015, D = 0.81 ± 0.62, d = 1.04) in EXP2.	Although outcomes of high-cognitive intervention support that it could improve cognitive flexibility, not statistically significant differences between groups highlighted the importance of future research.
			EXP2 (n = 29) Low-cognitive group.						
			CON (n = 19) Habitual PE classes.						
Takehara et al. [30]	To evaluate the effects of an exercise-based intervention on children’s academic achievement	Nº children: 2101. Schools: 10 Country: Mongolia. Age: 9−10.	EXP (n = 1069) High-intensity interval exercise program.	EXP did high-intensity interval exercise program while CON did usual physical educational classes.	10 weeks 10−25 min per session.	Flanker test.	Cognitive function.	Between group comparison did not reveal any significant difference on cognitive functions.	High-intensity interval training did not show better strategy than usual PE classes.
			CON (n = 1032) Usual PE classes.						
Kolovelonis & Goudas [31]	To evaluate the effects of different intervention programs on executive functions.	Nº children: 140. Schools: 4. Country: Greece. Age: 11.	EXP1 (n = 29) PA games highlighting contextual interference.	EXP1 did PA games highlighting contextual interference, EXP2 did PA games highlighting mental control, EXP3 PA games highlighting discovery did, and CON did not participate in physical education.	3 45-min sessions per week.	Design fluency test.	Cognitive flexibility.	Children from all EXP groups showed significative positive effects on executive functions compared to CON, although no differences between three EXP groups was observed.	The study supports the implementation of cognitive challenging PA games to foster executive functions.
			EXP2 (n = 36) PA games highlighting mental control.						
			EXP3 (n = 36) Physical activity games highlighting discovery.						
			CON (n = 39) Without PE.						
Sánchez-López et al. [34]	To analyze the effects of a multicomponent intervention on schoolchildren’s cognition.	Nº children: 240. Schools: 9. Country: Spain. Age: 5−7.	EXP (n = 82) Movi-Kids intervention.	EXP did Movi-Kids intervention while CON did usual physical educational classes.	3 sessions weekly 80 sessions 60 min sessions	Battery of General and Differential Aptitudes.	Cognition changes.	When compared with CON, children in EXP experimented significant higher improvements after intervention than in pretest (*p* ≤ 0.05) (effect size ranged from 0.33 to 1.48).	The intervention program was more effective than habitual PE classes for improving cognitive-related variables.
			CON (n = 158) Habitual PE classes.						
Lakes & Hoyt. [38]	To evaluate the influence of tae kwon do-based program on math performance.	Nº children: 200. Schools: 1. Country: United States. Age: 10−11.	EXP (n = 100) Martial arts intervention (LEAD).	EXP did martial arts intervention (LEAD) while CON habitual PE classes.	3 months 2−3 times per week.	WISC-III.	Cognitive self-regulation.	Children in the martial arts group showed greater self-regulation in response to a challenge than children in the CON for all three dimensions of self-regulation [Fs(1,174) = 11.18, 7.38, and 3.93, ps < 0.05 for cognitive, affective, and physical self-regulation, respectively]. Boys in EXP improved more their self-regulation than those in CON.	LEAD intervention is suitable for improving self-regulation.
			CON (n = 100) Habitual PE classes.						
**Academic performance**									
García-Hermoso et al. [18]	To evaluate before school exercise program on school children academic performance.	Nº children: 171. Schools: 3. Country: Chile. Age: 8−10.	EXP (n = 100) Active-Start.	EXP did Active-Start program while CON did usual PE classes.	8 weeks 5 times per week.	Children’s grades in the core subjects (mathematics and language).	Academic performance.	The children in EXP experimented improvements in both language (0.63; 95% CI 0.49 to 0.77) and mathematics (0.49; 95% CI 0.32 to 0.66) performance (*p* < 0.001).	Cardiorespiratory fitness through PA before school may fuel academic performance.
			CON (n = 70) Usual PE.						
Westendorp et al. [22]	To evaluate the effects of ball-skills-based program on cognitive parameters of children with learning disorders.	Nº children: 91. Schools: 1. Country: The Netherlands. Age: 7−11.	EXP (n = 45) ball skill intervention.	EXP did ball skill intervention program while CON did usual PE classes.	16 weeks 2 times per week 40 min each session.	TMT Dutch World in Numbers test.	Academic achievement.	Regarding cognitive related parameters, no intervention effects were found on the cognitive parameters. Further, no significant correlations were found between the changes in TOL performance (creativity test) and the changes in learning lags on reading and mathematics (all *p* values > 0.05) during the intervention or six months later.	The ball skill intervention program did not show positive effects on academic performance
			CON (n = 46) Habitual PE classes.						
Pinto-Escalona et al. [41]	To examine the effects of a school-based karate intervention on academic achievement.	Nº children: 721. Schools: 20. Country: Spain, Portugal, France, Poland. Age: 7−8.	EXP (n = 388) School-based karate intervention.	EXP did school-based karate intervention program while CON did usual PE classes.	1 year 2 h per week.	Curriculum evaluation criteria.	Academic performance.	Compared to CON, children in EXP improved significantly their academic achievement (d = 0.16; *p* = 0.003).	The study supports the use of martial arts-based program to enhance academic performance
			CON (n = 333) Habitual PE classes.						
De Bruijn et al. [25]	To evaluate the effects of different school-based PE programs on children’s academic performance-related variables.	Nº children: 891. Schools: 22. Country: The Netherlands Age: 7,4-11,14.	EXP1 (n = 221) Aerobic intervention.	EXP1 did aerobic intervention, EXP2 did cognitively engaging program, and CON did a program based on low cognitive and low physical exertion.	14 weeks 4 lessons per week.	Reading and mathematics tests provided by the government. Spelling test.	Academic achievement Reading performance Mathematic performance	Children with lower performance in reading at baseline performed better in reading at the post-test in the EXP2 group than in the CON (β = −0.06 (0.03), *p* = 0.03, 95% CI [−0.11 to −0.01]). No significant relation was found for the interaction between the dummy variable contrasting the EXP2 and the CON and baseline mathematics performance (β = −0.03 (0.04), *p* =.37, 95% CI [−0.11 to 0.04]), or baseline spelling performance (β = 0.07 (0.04), *p* = 0.06, 95% CI [−0.01 to 0.14]).	This study found no significant effects of two PA interventions on academic achievement, a conclusion that corroborates existing literature in which mixed findings on the effectiveness of PA are reported.
			EXP2 (n = 240) Cognitively-engaging program.						
			CON (n = 430) Habitual PE classes.						
Sallis et al. [27]	To evaluate a PE program on academic achievement.	Nº children: 754. Schools: 12. Country: United States. Age: 9.	EXP (n = 330) SPARK.	EXP did SPARK program while CON did usual PE classes.	2 years 4 weeks 3 days per week.	Metropolitan Achievement Tests.	Language performance. Mathematics performance. Reading performance.	Match score: no effects Language score: the decline in percentile ranking was significantly less in the Trained Teacher condition than in the CON. Reading score: students in the EXP significantly increase in percentile ranking while the CON declined.	The results suggested that engaging children in more time of physical not necessarily influence academic performance.
			CON (n = 225) Habitual PE classes.						
Aadlamd et al. [28]	To evaluate the effects of curriculum prescribed program on executive functions.	Nº children: 1202. Schools: 57. Country: Norway. Age: 10.	EXP (n = 596) Active Smarter Kids.	EXP did Active Smarter Kids intervention while CON did usual PE classes.	7 months 135 min per week of PA.	Verbal fluency test WISC-IV.	Verbal fluency.	Children in EXP did not show significant effects on participants verbal fluency (the mean group difference for verbal fluency was 0.17 (−0.31 to 0.65), with a *p*-value of 0.484 in the completers-only analysis).	The evaluated program is not effective for improving children’s verbal fluency.
			CON (n = 533) Habitual PE classes.						
Takehara et al. [30]	To evaluate the effects of an exercise-based intervention on children’s academic achievement.	Nº children: 2101. Schools: 10 Country: Mongolia. Age: 9−10.	EXP (n = 1069) Intervention program.	EXP did intervention program while CON did usual PE classes.	10 weeks 10–25 min per session.	Mongolian national language scores. Flanker test.	Academic achievement. Cognitive function.	Compared to children in CON, the program performed by children in EXP improves: total examination scores, mathematics scores, and Mongolian language scores. When compared to CON, children in EXP recruited from urban areas showed an 8.36-point improvement (95% CI: 6.06 to 10.66) in academic scores, whereas those in a mixed residential area showed a 9.55-point improvement (95% confidence interval: 6.58 to 12.51).	The program performed by EXP is suitable for fueling academic achievement.
			CON (n = 1032) Control group.						
De Fano et al. [33]	To evaluate the effects of enriched PE program on children fluency and flexibility.	Nº children: 242. Schools: 2. Country: Italy. Age: 10−11.	EXP (n = 129) Past enriched PE.	EXP did past enriched PE while CON did usual PE classes.	6 months 1 time per week. 1 h.	RNG task GPAI.	Fluency. Flexibility.	*Fluency* The results showed significant interaction between CON and EXP was found for fluency (F(1, 134) = 4.74, *p* = 0.048) and switching (F(1, 165) = 4.69, *p* = 0.032). *Flexibility* Compared to the children in CON at the end of the intervention, children in EXP showed higher scores (Cohen’s d = 0.46) and lower originality values (−0.33).	The results showed positive effects on fluency and flexibility with continued participation in the program.
			CON (n = 112) Past conventional PE.						
Pinto-Escalón et al. [41]	To evaluate the effects of Karate-based program on children’s academic achievement.	Nº children: 388. Schools: 20. Country: Spain, Portugal, France, Germany, and Poland. Age: 7−8.	EXP (n = 388) Karate Mind and Movement program.	EXP did Karate Mind and Movement program.	1 year 2 h per week.	GPA.	Academic performance.	Responders for the SDQ presented higher SDQ scores (i.e., higher psychosocial difficulties) at baseline than non-responders (*p* < 0.001). Responders for academic performance were mostly males (*p* = 0.017), with an older age (*p* = 0.030), and with worse academic performance (*p* < 0.001) at baseline compared with non-responders, and tended to present higher SDQ scores (*p* = 0.055). Responders for one outcome obtained greater benefits from the EXP on the other outcome (e.g., responders for SDQ improved academic performance [*p* < 0.001] compared with non-responders).	Although the results of CON were not included in the statistical analysis, EXP seems particularly effective for children with psychosocial difficulties and low academic performance.
			CON (n = not included in the analysis).						
Telford et al. [36]	To evaluate the effects of specialist-taught PE on academic development.	Nº children: 620. Schools: 13. Country: Australia. Age: 8−9.	EXP (n = 312) Specialist-taught PE.	EXP did specialist-taught PE while CON did common-practice PE.	2 years 150 min per week.	Local government education tests.	Writing proficiency. Numeracy proficiency. Reading proficiency.	While no evidence was found on children’s reading effects, children in EXP improved greater on numeracy (*p* < 0.03) and writing (*p* = 0.13) scores.	The results support the role of PE in academic development.
			CON (n = 308) Habitual PE classes.						
Lakes & Hoyt. [38]	To evaluate the tae kwon do-based program on math performance.	Nº children: 200. Schools: 1. Country: United States Age: 10−11.	EXP (n = 100) Martial arts intervention (LEAD).	EXP did martial arts intervention (LEAD) while CON habitual PE classes.	3 months. 2–3 times per week.	WISC-III.	Math performance.	Compared with CON, children in EXP improved performance on a mental math test.	The martial arts based program seems suitable for improving match performance.
			CON (n = 100) Habitual PE classes.						
Katz et al. [39]	To assess the effects of a PA-based program on health-related outcomes.	Nº children: 1214. Schools: 5. Country: United States. Age: 7–10.	EXP (n = 655) Activity Bursts in the classroom	EXP did activity bursts in the classroom intervention while CON normal curricular activities.	1 year 30 min per day.	Missouri Academic Performance scores.	Academic performance.	Based on MAP achievement level scores: no significant differences between groups in reading (*p* = 0.35) and mathematics (*p* = 0.15). Based on ISD progress reports: in mathematics (28.6% vs. 20.8%, *p* < 0.001) and reading (21.1% vs. 16.1%, *p* = 0.01) a greater proportion of students whose academic performance improved compared to that of the EXP.	The results did not show that the intervention program was more suitable than habitual PE classes for improving academic performance.
			CON (n = 559) Normal curricular activities.						
					**Creativity**				
Westendorp et al. [22]	Evaluate the effects of a program based on ball skills on primary school children’s problem-solving ability.	Nº children: 91 (with learning disorders). Schools: 1. Country: The Netherlands. Age: 7−11.	EXP (n = 45) ball skill intervention.	EXP did ball skill intervention program while CON did usual PEclasses.	16 weeks 2 times per week 40 min each session.	Tower of London test.	Problem solving skills.	The intervention group showed changes in problem-solving mainly in those who showed larger improvements in ball skills (r = 0.41, *p* = 0.007).	Implementing activities for improving ball skills may help for enhancing problem-solving abilities.
			CON (n = 46) Habitual PE classes.						
Ángel Latorre-Román et al. [23]	Assess the effects of active recess-based program on schoolchildren ability.	Nº children: 114. Schools: 3. Country: Spain. Age: 8−12.	EXP (n = 58) Active Recess Programme.	EXP did Active Recess program while CON did habitual class activities with no physical exercise.	10 weeks 3 times per week 30 min each activity.	PIC-N.	Creativity.	The EXP group showed significant improvements showed larger increases in EXP than in CON in creativity (CON = *p* < 0.05; EXP = *p* < 0.001).	Active Recess based strategies are suitable for improve creativity.
			CON (n = 56) Habitual activities with no physical exercise.						
De Fano et al. [33]	Analyze the effects of a program on schoolchildren’s different abilities.	Nº children: 242. Schools: 2. Country: Italy. Age: 10−11.	EXP (n = 129) Past enriched PE.	EXP did past enriched PE while CON did usual PE classes.	6 months 1 time per week 1 h.	GPAI.	Originality.	EXP showed significant differences (F(1, 165) = 5.56, *p* = 0.019; ES of Cohen’s d = −0.33). Children in EXP showed better values in originality than CON.	Enriched PE classes are more suitable for enhancing originality, at least in short term.
			CON (n = 112) Past conventional PE.						
**Others**									
De Bruijn et al. [21]	Compare the effects of different programs on brain activation.	Nº children: 92. Schools: 22. Country: The Netherlands. Age: 8−10.	EXP1 (n = 30) Aerobic group.	EXP1 did aerobic program, EXP2 did cognitively engaging program, and CON did a program based on low cognitive and low physical exertion.	30 min 4 lesson per week 14 week.	The Spatial Span tasks.	Brain activation.	Pretest: no significant differences between groups on the spatial span task (F (2, 59) = 0.15, *p* = 0.86). Post-test: in general, significant differences between pretest and post-test were found (F (1, 59) = 12.32, *p* < 0.001). However, brain activation was no significantly differ between both intervention groups.	Both aerobic group and cognitive engaging group are suitable for enhance brain activation.
			EXP1 (n = 31) Cognitively engaging group.						
			CON (n = 31) Habitual PE classes.						

Note: ASK = Active Smarter Kid; AWMA = Automated Working Memory Assessment; CON = control group; EXP = experimental group; CI = confidence interval; DCSS = dimensional change card sort; GPAI = game performance assessment instrument; MVPA = moderate-to-vigorous intensity level; PA = physical activity; PE = physical education; PF = physical fitness; PIC-N = creative imagination test; RAVLT = Rey Auditory Verbal Learning Test; RNG = random number generation task; SDQ = strengths and difficulties questionnaire; StroopCW = Stroop Color Word; TEA = school aptitude test; TMT = trail making test; VO2 = maximal oxygen uptake; WISC-III = Wechsler Intelligence Scale for Children third edition; WISC-IV = Wechsler Intelligence Scale for Children fourth edition.

## Supplementary Materials

The characteristics of included intervention programs can be downloaded at: https://www.mdpi.com/article/10.3390/sports13080261/s1.
