# Supplementary material for: The Effect of Physical-Activity-Based Programs on School Children’s Cognitive Competence-Related Variables: A Systematic Review of Randomized Controlled Trials
Source: Sports (Basel). 2025 Aug 8;13(8):261. doi: 10.3390/sports13080261 (PMC12390218; doi:10.3390/sports13080261)
Supplement: Supplementary file 1 [file sports-13-00261-s001.zip › sports-3746629-supplementary.pdf]

Table S1. *Intervention programs` details*

Reference	Name of the program	Main ideas
García-Hermoso et al. [18]	Active-Start	The Active-Start is mainly a program of cooperative physical games, which have been structured to make group cooperation essential to game success and to encourage pro-social skills. The games challenge and encourage children's resilience while requiring cooperation to succeed, due to success is not determined on an individual basis, but rather as an overall group success. The intervention program also included sports games adapted to the age of the participants, playground games, dance, and other recreational activities.<br><br>The intensity of the main part of the sessions was moderate-to-vigorous according to a previous study. Also, different activities favoring social interactions were performed to facilitate the interactions between the participants at the end of the sessions (cool-down).
Chou et al. [19]	Movement Games Intervention	The overweight children in the intervention group, along with their classmates, participated in the movement games.<br><br>For the intervention, movement games were designed based on the movement concept and object manipulation skills, and the games were modified.<br><br>Movement concepts and skills focused on the ability to move in various situations, respond to speed, direction,and force of movements, and control body movements while jumping, throwing, catching, dribbling, kicking, or passing. During the movement games, PE teachers educated the intervention group's overweight children in a variety of cognition-engaging movement skills with comprehension and intensity relevant to the program requirements. With the movement games, the goal in this intervention was to help enhance eye–hand coordination with reaction time and cognitive processing speed.<br><br>Each lesson began with a 10-min warm-up (moderate cardiovascular activity and static and dynamic stretching), followed by a 30-min movement game of hand coordination with reaction time and cognitive processing speed.
Schmidt et al. [20]	Team Games	This intervention consisted of specifically designed team games (floorball and basketball) tailored to challenge s.

		First, these two team games were chosen because according to ACSM exercise guidelines American College of Sports Medicine they are appropriate to induce moderate to vigorous PA intensity, which should promote aerobic fitness when performed regularly.
		Second, both team games contain large amounts of prospective control and complex eyehand coordination, and require goal-directed behavior.
		Third, these team games were suitable for combining sport-specific skill development (as required by the curriculum) with enriched cognitive engagement.
	Aerobic Exercise	This condition consisted of different group-oriented and playful forms of aerobic exercises, whose main aim was to promote children's aerobic fitness. Although it is not possible to exclude cognitive engagement entirely from chronic PA interventions, the attempt was made to choose exercises that were not cognitively demanding.
Schmidt et al. [20]	Cognitively Demanding Physical Education Lesson	This type of exercise was chosen because it was postulated that coordinatively demanding and non-automated sport-related activities activate the same brain regions that are used to control higher-order cognitive processes. Based on this theoretical assumption of shared information processes in both motor and cognitive control, cognitively demanding physical activities should require specific higher-order cognitive processes, such as executive functions (with focused attention being a part of inhibitory control), and should further activate them for subsequent use.

De Bruijn et al. [21]	Cognitively-engaging Intervention	The cognitively-engaging intervention focused on challenging children's cognitive and motor skills by including exercises (e.g. throwing and catching, balancing) and games (e.g. soccer and dodgeball) that required complex movements, and that engaged children's cognitive skills via difficult or fast-changing rules.
	Aerobic Physical Activity Intervation	Intervention focused on aerobic physical activity, timing to improve children's cardiovascular fitness via exercises that elicited high heart rate levels. Included exercises focused on repetitive and automated skills, for example running, relays, or individualized exercises such as jumping jacks, planks, or squats.
Westendorp et al. [22]	Ball Skill Intervention	Ball skills were first practiced in more simple, static settings with simple exercises like throwing and catching with two children or bouncing and turning around cones. The simple exercises in static settings were aimed at an adequate development of basic ball skills (i.e., automatization of ball skills).<br><br>Later on, the tasks became more complex such as throwing, catching, and bouncing during a ball game, where children needed to pay attention to teammates, opponents, game rules, and time, which required more cognitive engagement than simple exercises.
Ángel Latorre-Román et al. [23]	Active Recess Program	The teachers were previously instructed in the training protocol about the exercises to be performed and the work and rest guidelines as well as the intensity of the motor tasks, i.e., the activities should take hard physical effort causing heavier than normal breathing and sweating.<br><br>The teachers provided positive feedback to the students and setting challenges in carrying out tasks.<br><br>All sessions included a HIIT which generally refers to repeated short to long bouts of high-intensity exercise interspersed with recovery periods. It was employed a HIIT such as small-side games (e.g., football, basketball), which require cooperation with other schoolmates, strategies coordination, and adaptation to continually changing task demands.
Pinto-Escalona et al. [41]	School-based Karate Intervention	The intervention provided children with sensory-motor stimuli for the development of basic motor skills and cognitive performance while facilitating collaboration. An enriched environment was created in school gyms using tatamis on the floor to ensure safety and non-hazardous modular materials to facilitate specific motor actions, with materials including sponge balls, hurdles, hoops, sticks, and cones.

		The main part of the session consisted of non-specific motor tasks aimed at improving cardiorespiratory fitness, strength, coordination, balance and flexibility (e.g., gymnastic exercises such as somersaults, balance tasks, and jumps), and also included some karate-specific motor skills.
De Bruijn et al. [25]	Aerobic Intervention	The focus of the aerobic intervention was on MVPA, aiming to elicit high heart rate levels to promote children's aerobic fitness via playful forms of aerobic exercise that were highly repetitive and automated, for example, relays, running or individual exercises such as doing squats.
	Cognitively-engaging Intervention	The cognitively engaging intervention focused on challenging cognition and motor skills via games (e.g., dodgeball and soccer) and exercises (e.g., balancing, throwing, and catching) that required complex coordination of movements, and that included complex and fastchanging rules to engage children's cognitive skills
Etnier et al. [26]	PACER	The PACER (Progressive Aerobic Cardiovascular Endurance Test) is a valid and reliable measure of aerobic capacity that is a part of the FITNESSGRAM. The test is progressive in that the intensity changes from easy to hard across the course of the test.
Sallis et al. [27]	Spark Program	SPARK is a comprehensive curriculum and professional development program designed to promote physical activity in and out of school. SPARK physical education classes are designed to promote high levels of physical activity that will improve health-related fitness, promote movement skills that add to success and enjoyment in physical activity, and encourage positive socialization.
Aadlamd et al. [28]	Active Smarter Kid	The ASK intervention was a part of the mandatory school curriculum for all children attending the intervention schools., (2) PA breaks during classroom lessons (5 min/school day), and (3) PA homework (10 min/school day).
		(1) Physically active educational lessons (3 × 30 min/week) in the subjects Norwegian, mathematics, and English
		(2) It was led by classroom teachers and consisted of three components, generating an additional 165 min/week of PA
		(3) PA homework (10 min/school day).
Oppici et al. [29]	High-cognitive program and Low-cognitive program	The two EXP practiced dance for 7 weeks, twice a week, learning a choreography, while the CON participated in the school standard PE curriculum. In the high-cognitive group, the dance teachers limited visual demonstrations and encouraged children to memorise and recall movement sequences to increase the cognitive challenge.
Takehara et al. [30]	Intervention program	For the intervention program, we adopted an HIIT-based exercise program combined with music. The program comprised 4 exercise parts separated by rest intervals. The exercise parts had various types of movements intended to improve not only aerobic fitness but also basic

		motor skills. We used music originally arranged and optimized to support dynamic and fluid movement of children. The music tempo was gradually increased to let exercise intensity increase incrementally.
Kolovelonis & Goudas. [31]	Physical activity games highlighting contextual interference	In this type of games contextual interference is created, that is the context and the conditions of the game change continuously during the game requiring students to make unpredictable sequences of actions.
	Physical activity games highlighting mental control	This type of games includes stopping games which require students to react in alternating signals to go and stop overriding prior actions, updating games which set memory demands for holding and manipulating information, and switching games which requires students to stop what they are doing and act in a totally different way.
	Physical activity games highlighting discovery	This type of games requires finding multiple solutions in problem solving conditions or in open-ended games, that is, games that the starting point, the rules, and the goal are explained, but not how to perform the game or what strategies to use.
Brocken et al. [32]	External focus instructions	The children in the external focus of attention groups were instructed "to move the golf club like a pendulum".
	Internal focus instructions	The children in the internal focus of attention groups were instructed "to move the arms like a pendulum.
De Fano et al. [33]	Past Enriched PE	Participants experienced the enriched PE along the primary school cycle, earlier or later, for a longer or shorter time according to the cross-over design. The first and second phase of the experience had a common core and some diversities to adapt them to the age-related PE goals. To specifically foster DMA and motor creativity, the intervention activities were taught with a Constraints-Led Approach and nonlinear pedagogical principles derived from an Ecological Dynamics conceptualization of PE. Teachers employed open-ended tasks, in which only the starting point, goal and rule(s) were indicated, and children were encouraged to find as many diversified solutions as possible. Cognitive engagement was generated by applying principles of variability of practice.

Sánchez-López et al. [34]	Movi-Kids	It consisted of traditional playground games and included three content blocks: sports team games, traditional games for the development of motor skills (simple tasks of balance, control of objects, jumps, and bilateral body coordination), and activities with music (games and simple choreographies).
Pinto-Escalón et al [24]	Karate Mind and Movement program	The intervention provided children with sensory-motor stimuli for the development of basic motor skills and cognitive performance while facilitating collaboration. The main part consisted of non-specific motor tasks aimed at improving cardiorespiratory fitness, strength, coordination, balance, and flexibility (e.g., somersaults, jumps, dynamic flexibility, advance in lunge position, going around vertical plastic sticks, and kicking to sponge balls). The final part of the sessions included stretching exercises, discussion about the class (e.g., feelings, difficulties), and final bows (see Pinto-Escalona et al. [24] for more details)
Van Den Berg et al. [35]	Intervention program	Educators developed and produced 20 instruction videos in which a juggler shortly introduced the juggling exercises and then combined the juggling exercises with the multiplication tables. The complexity of the juggling exercises increased each week, i.e., practicing with easy throw and catch exercises with one ball in week 1 and 2, two balls in week 3 and 4, and ending with using three balls in week 5 of the program.<br><br>Children were instructed to answer the multiplication table sums given by the juggler verbally when they caught the ball. A fixed rhythm was used for throwing and catching the balls and answering the math sums. The videos were displayed on digital screens in the classrooms while children were standing and juggling behind their desks.
Telford et al. [36]	Specialist-Taught	The specialist-taught PE lessons devoted a significantly larger median percentage of lesson time to activities related to fitness, including strength, flexibility, and static and dynamic postural activities (17.6% for specialist-taught PE vs 2.1% for common-practice PE; $P < .001$).<br><br>The specialist teachers spent a greater median percentage of the lesson personally demonstrating and participating in fitness-related activities.<br><br>The specialist teachers (but not the common-practice teachers) emphasized development of posture, balance, and breathing control through a variety of yogalike static and dynamic activities, which often required muscular strength. The specialist teachers always participated in activities, whereas common-practice teachers usually did not.

		The specialist teachers (but not the common-practice teachers) consistently encouraged individual and group discussions of game and skill development strategies and introduced quiet periods of reflection at the end of the lesson.
Lakes & Hoyt [38]	LEAD	Students were told to line up in a particular formation and stand at attention. Students then faced the instructor and bowed to demonstrate respect. At the start of each class, students spent a few minutes sitting in meditation. Students were instructed to clear their minds of thoughts and worries and to focus solely on their breathing. Deep-breathing techniques were taught and reinforced during meditation.<br><br>Common to the teaching of all techniques was the principle that to progress, the student should ask himself/herself three questions designed to promote self-monitoring: (1) Where am I? (2) What am I doing? (3) What should I be doing? After asking themselves these questions, students were told to correct their stances, behavior, or thoughts, thus regulating their behavior and thoughts in accordance with the expectations of the particular situation. The instructor emphasized that students were responsible for their own behavior, not only in the LEAD.
Katz et al. [39]	Activity Bursts in the classroom	The program was flexible; activity intensity could be raised or lowered according to the athletic abilities and attention spans of the students. The program was intended as a supplement to physical education programs. The cognitively demanding exercise intervention consisted of team games or exercises that require complex coordination of movements, strategic play, cooperation between children, anticipating on the behavior of teammates or opponents, and dealing with changing task demands. Each activity burst had 3 components:
		1) a warm-up that could include stretching or light aerobic activity (eg, walking, arm circles, muscle stretching).
		2) a core activity consisting of strength activities or aerobic activities (e.g. hopscotch, lunges, squats, star jumps, jogging, walking quickly, hopping, dancing to music, skipping)
		3) A cooldown similar to warm-up activities, consisting of stretching or low-intensity activity.
Maijer et al. [40]	Aerobic exercise	The lessons consisted of a warm-up phase of 10 min and a core phase of 20 min. The aerobic exercise intervention consisted of activities specifically designed to target moderate-to-vigorous intensity while avoiding high cognitive demands. The focus was on highly repetitive and automated exercises, such as circuit training, relay games, playing tag, and individual activities like running or doing squats.

	Cognitively demanding exercise	The cognitively demanding exercise intervention consisted of team games or exercises that require complex coordination of movements, strategic play, cooperation between children, anticipating on the behavior of teammates or opponents, and dealing with changing task demands. Each activity burst had 3 components.

Note: HIIT = high intensity interval training; MVPA = moderate to vigorous physical activity; PA = physical activity; PE = physical education
